# Altering the levels of nuclear import factors in early *Xenopus laevis* embryos affects later development

**DOI:** 10.1371/journal.pone.0215740

**Published:** 2019-04-22

**Authors:** Predrag Jevtić, Richik N. Mukherjee, Pan Chen, Daniel L. Levy

**Affiliations:** Department of Molecular Biology, University of Wyoming, Laramie, Wyoming, United States of America; University of Colorado Boulder, UNITED STATES

## Abstract

More than just a container for DNA, the nuclear envelope carries out a wide variety of critical and highly regulated cellular functions. One of these functions is nuclear import, and in this study we investigate how altering the levels of nuclear transport factors impacts developmental progression and organismal size. During early *Xenopus laevis* embryogenesis, the timing of a key developmental event, the midblastula transition (MBT), is sensitive to nuclear import factor levels. How might altering nuclear import factors and MBT timing in the early embryo affect downstream development of the organism? We microinjected *X*. *laevis* two-cell embryos with mRNA to increase levels of importin α or NTF2, resulting in differential amounts of nuclear import factors in the two halves of the embryo. Compared to controls, these embryos exhibited delayed gastrulation, curved neural plates, and bent tadpoles with different sized eyes. Furthermore, embryos microinjected with NTF2 developed into smaller froglets compared to control microinjected embryos. We propose that altering nuclear import factors and nuclear size affects MBT timing, cell size, and cell number, subsequently disrupting later development. Thus, altering nuclear import factors early in development can affect function and size at the organismal level.

## Introduction

The nucleus plays many important roles in the cell. The nuclear envelope (NE) is composed of a double lipid bilayer. The outer nuclear membrane is continuous with the endoplasmic reticulum while the inner nuclear membrane is lined and supported by the nuclear lamina, composed of a meshwork of lamin intermediate filaments and lamin-associated proteins [1, 2]. Nuclear pore complexes (NPC) that mediate nucleocytoplasmic transport are inserted into the NE at sites where the inner and outer nuclear membranes fuse [2–5]. After mitosis and nuclear reassembly, lamins are imported into the nucleus along with other proteins containing nuclear localization signals (NLS). Classical nuclear import is mediated by importin α/β karyopherins, which bind NLS-containing proteins and ferry them across the NPC and into the nucleus. Within the nucleus, Ran in its GTP-bound state binds to importin β thereby releasing NLS cargos. Another key player in this process is NTF2, a dedicated nuclear import factor for Ran [6–10]. Associated with the NPC, NTF2 has been shown to reduce import of large cargos [11–13]. While nuclear import is critical for a wide variety of cell functions [14, 15], in this study we investigate how altering the levels of nuclear import factors impacts developmental progression and organismal size.

In *Xenopus*, levels of two nuclear import factors were shown to tune rates of nuclear import, which coincidentally also impacted nuclear growth. Increased importin α levels generally positively scale with nuclear import and size while increased NTF2 negatively regulates import of large cargos and nuclear size, although differential effects are observed when these factors are present at very high levels and depending on the cellular context [12, 13]. Nuclear lamins represent one imported cargo that contributes to nuclear growth [16]. How might nuclear import impact development? During early *X*. *laevis* development, the first twelve cleavage cell divisions occur rapidly with little new transcription occurring (i.e. stages 1–8). Stage 8 coincides with the midblastula transition (MBT) when zygotic transcription is upregulated, cell cycles slow, and there is onset of cell division asynchrony and motility [17–20]. While the DNA-to-cytoplasm ratio is one important factor that determines when the MBT initiates [17, 18, 21–23], we previously demonstrated that altering nuclear import factor levels and nuclear size in early *X*. *laevis* embryos also affects MBT timing [24, 25]. An important question raised by these studies is how altering nuclear import factors and MBT timing in the early embryo affects downstream development of the organism.

Here we test how altering levels of nuclear import factors in the early embryo affects later development. Previous work has shown how levels of importin α and NTF2 impact nuclear import in *Xenopus* embryos and mammalian cell culture [12, 13, 26–29]. It has also been shown that the levels of NTF2 inversely correlate with nuclear enlargement during melanoma progression and that NTF2 overexpression was sufficient to reduce nuclear size in primary melanoma [13]. There is growing evidence that early embryogenesis and cancer progression share similar cellular features and that many embryo-specific genes and signaling pathways are reactivated in cancer [30, 31]. For these reasons, we were particularly interested to test the developmental consequences of altering NTF2 levels because of its potential involvement in carcinogenesis [13, 32, 33]. In this study, we investigate how altering the levels of nuclear import factors in *X*. *laevis* embryos impacts gastrulation, neurulation, and development of tadpoles and froglets.

## Results and discussion

**Fig 1:**
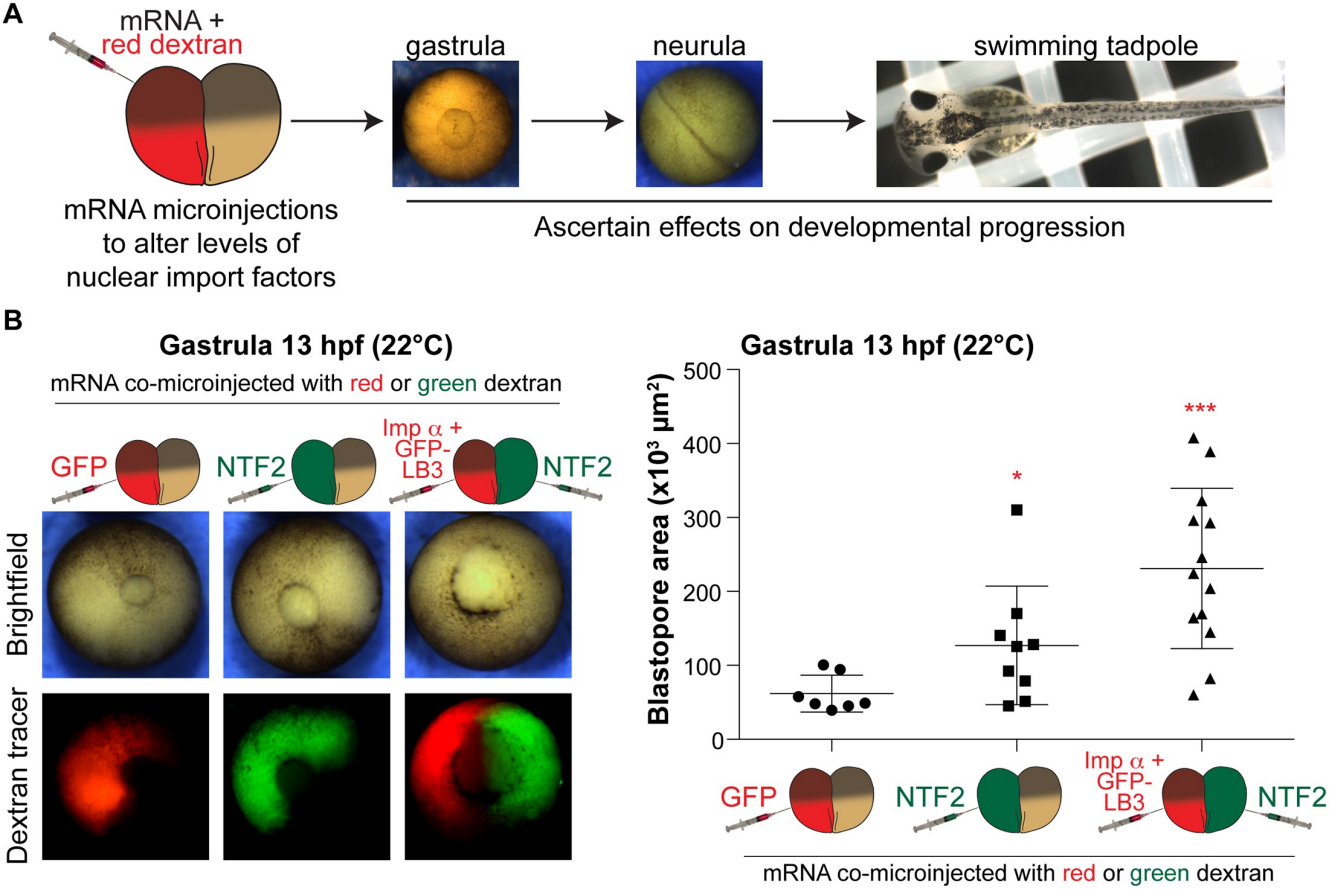
Differential levels of nuclear import factors in the two halves of an early embryo affect gastrulation timing. **(A)** Experimental approach. One blastomere of a two-cell stage *X*. *laevis* embryo was co-microinjected with mRNA to alter nuclear import factor levels and fluorescently labeled dextran as a cell tracer. Embryos were allowed to develop to different stages to assess effects on developmental progression. **(B)** Microinjections were performed as shown in (A) with 250 pg GFP mRNA, 175 pg NTF mRNA, or 250 pg importin α mRNA + 250 pg GFP-LB3 mRNA. These amounts, that maximally affect nuclear size [13, 24], were used in all experiments. Microinjected two-cell embryos were allowed to develop to 13 hpf gastrula. Representative vegetal pole images are shown. Blastopore area was measured and averaged for 7–13 embryos per condition. Blastopore closure appeared symmetric in all conditions. Error bars represent SD. *** p<0.005, * p<0.05.

We microinjected one blastomere of two-cell stage embryos with mRNA encoding nuclear import factors along with fluorescent dextran to trace cells that received the mRNA (Fig 1A). One important advantage of this approach is that the uninjected half of the embryo serves as an internal control, thus facilitating the observation of any developmental differences between the two halves of the embryo. In some cases we differentially microinjected the two blastomeres to maximize potential nuclear import differences in the two halves (i.e. importin α/lamin B3 in one half and NTF2 in the other half). For control experiments, embryos were microinjected with mRNA encoding either GFP or histone H2B-GFP. Embryos were microinjected with mRNA amounts previously shown to maximally alter nuclear size in vivo (S1 Fig) [12, 13, 24]. Because importin α and NTF2 are essential, we felt that overexpression of these factors would be more informative than knockdown, which might have had pleiotropic effects due to reduced cell and embryo viability.

To quantify the timing of gastrulation, we measured blastopore size in microinjected embryos 13 hours post fertilization (hpf). We previously showed that increasing importin α and lamin B3 (LB3) expression levels led to premature onset of the MBT and accelerated blastopore closure during gastrulation [24]. Conversely, NTF2 microinjection delayed blastopore closure, and an even greater delay was observed when half the embryo was microinjected with NTF2 and the other half was microinjected with importin α/LB3 (Fig 1B). These data suggest that different levels of nuclear import factors in the two halves of the embryo affect the timing of gastrulation.

**Fig 2:**
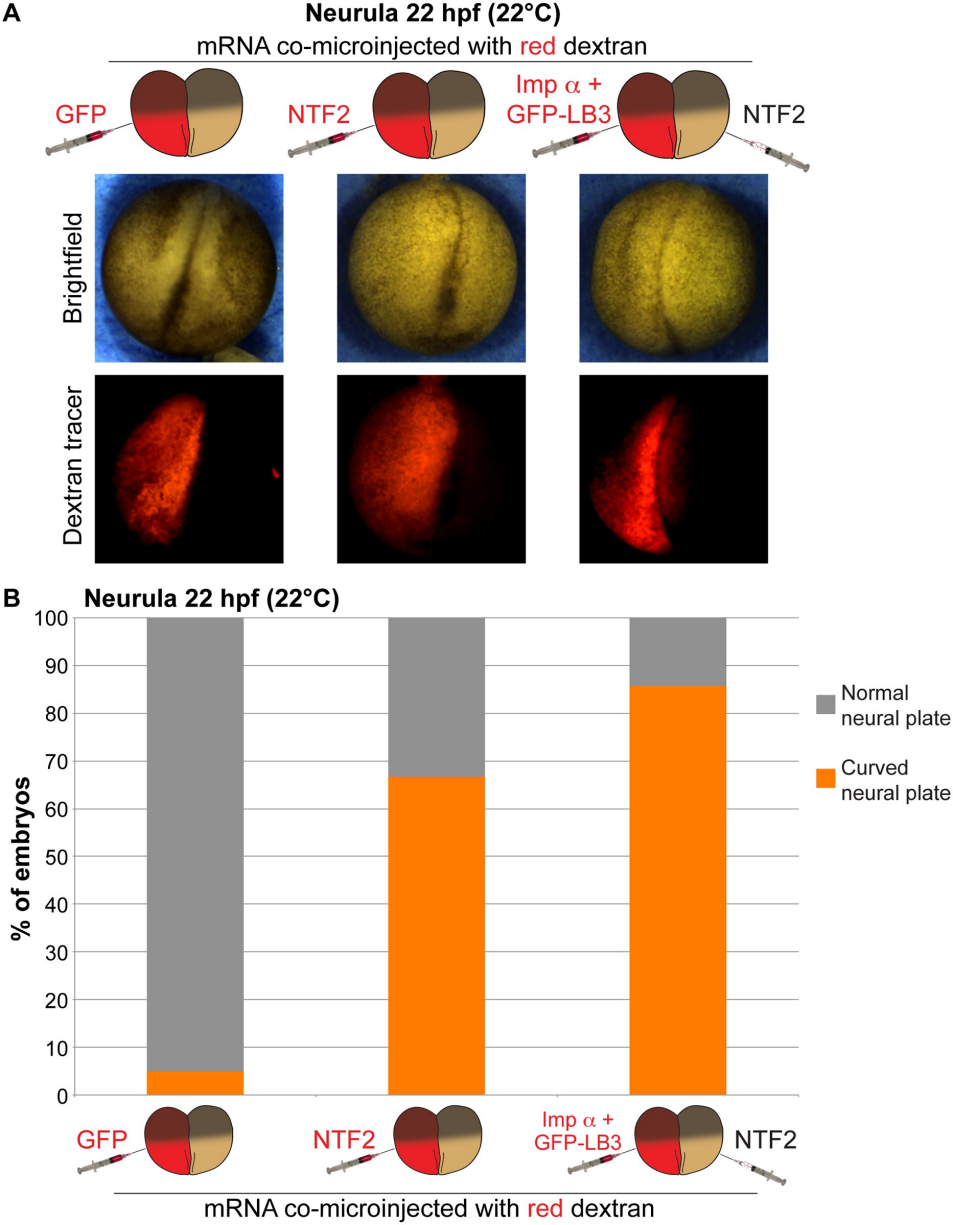
Differential levels of nuclear import factors in the two halves of an early embryo lead to neural plate curvature. **(A)** Two-cell embryos were microinjected as indicated and allowed to develop to 22 hpf neurula. Representative images are shown. **(B)** Neurula were scored as having normal or curved neural plates by drawing a line through the middle of the embryo. Embryo numbers: n = 19 for GFP, n = 21 for NTF2, n = 7 for imp α + GFP-LB3/NTF2.

We next examined how neurulation was affected when nuclear import factor levels were manipulated early in development. Altering levels of nuclear import factors in half of the embryo frequently resulted in differential timing of neural plate closure in the two halves of the embryo (S2A Fig and S1 Video). Consequently, these embryos exhibited a curved neural plate (Fig 2A, S3A Fig and S2 and S3 Videos). This phenotype was observed in over 65% of NTF2-microinjected embryos, increasing to 85% for embryos microinjected to maximize the import differential in the two halves of the embryo (Fig 2B). Similar phenotypes were observed when nuclear import factor mRNA was co-microinjected with H2B-GFP mRNA instead of dextran tracer and with frogs and embryos derived from two different frog colonies (S3A and S3B Fig). Furthermore, neuronal marker staining was similar on the two sides of bent neural plates (S3C Fig). Microinjection of importin α alone induced a similar effect as importin α/LB3 (Fig 2 and S3 Fig), and the formation of curved neural plates was dependent on sufficiently altering nuclear import factor levels as microinjecting lower amounts of NTF2 mRNA (e.g. 50 pg) failed to induce neural plate bending. When one-cell embryos were microinjected we observed no effect on the morphology of the neural plate compared to controls, demonstrating that the curved phenotype was dependent on there being differential amounts of nuclear import factors in the two halves of the embryo (S3B Fig).

Interestingly, the neural plate generally curved toward the NTF2 injected side or away from the importin α/LB3 injected side (Fig 2A and S3 Fig). Altering MBT timing and the onset of longer cell cycles has been shown to indirectly impact cell size [24, 34–36]. In particular, in the half of the embryo with increased importin α levels and nuclear size, early onset of longer cell cycles results in larger cells, potentially explaining why the neural plate curved away from that side of the embryo. Indeed, consistent with these previous reports, surface imaging showed smaller cells on the NTF2-injected side and larger cells on the importin α-injected size (S2B and S2C Fig).

**Fig 3:**
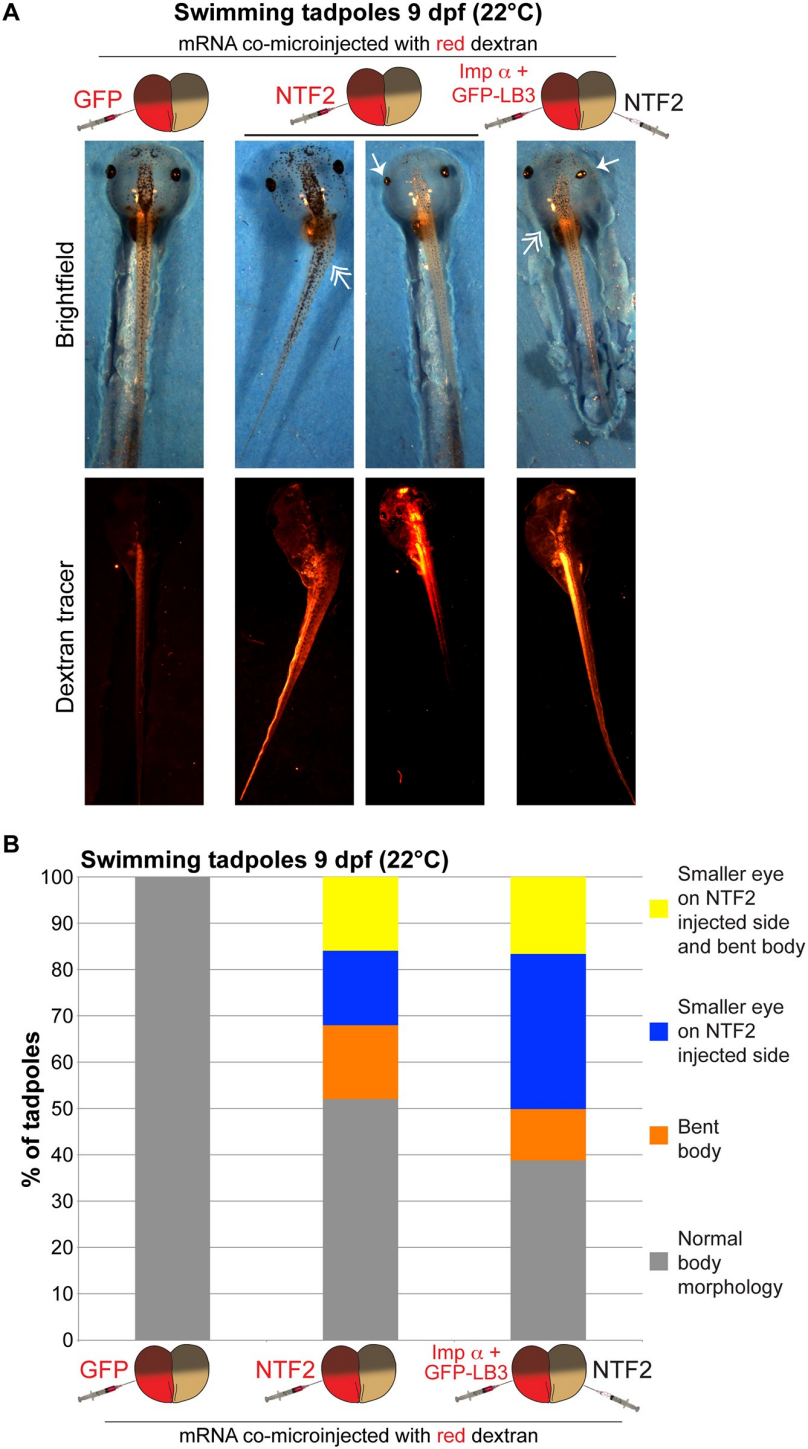
Differential levels of nuclear import factors in the two halves of an early embryo lead to bent tadpoles. **(A)** Two-cell embryos were microinjected as indicated and allowed to develop into 9 dpf swimming tadpoles. Representative images are shown. Single-headed arrows indicate small eyes. Double-headed arrows indicate bent bodies. **(B)** Tadpoles were scored as indicated by measuring eye areas and body axis angles. Embryo numbers: n = 10 for GFP, n = 25 for NTF2, n = 18 for imp α + GFP-LB3/NTF2.

**Fig 4:**
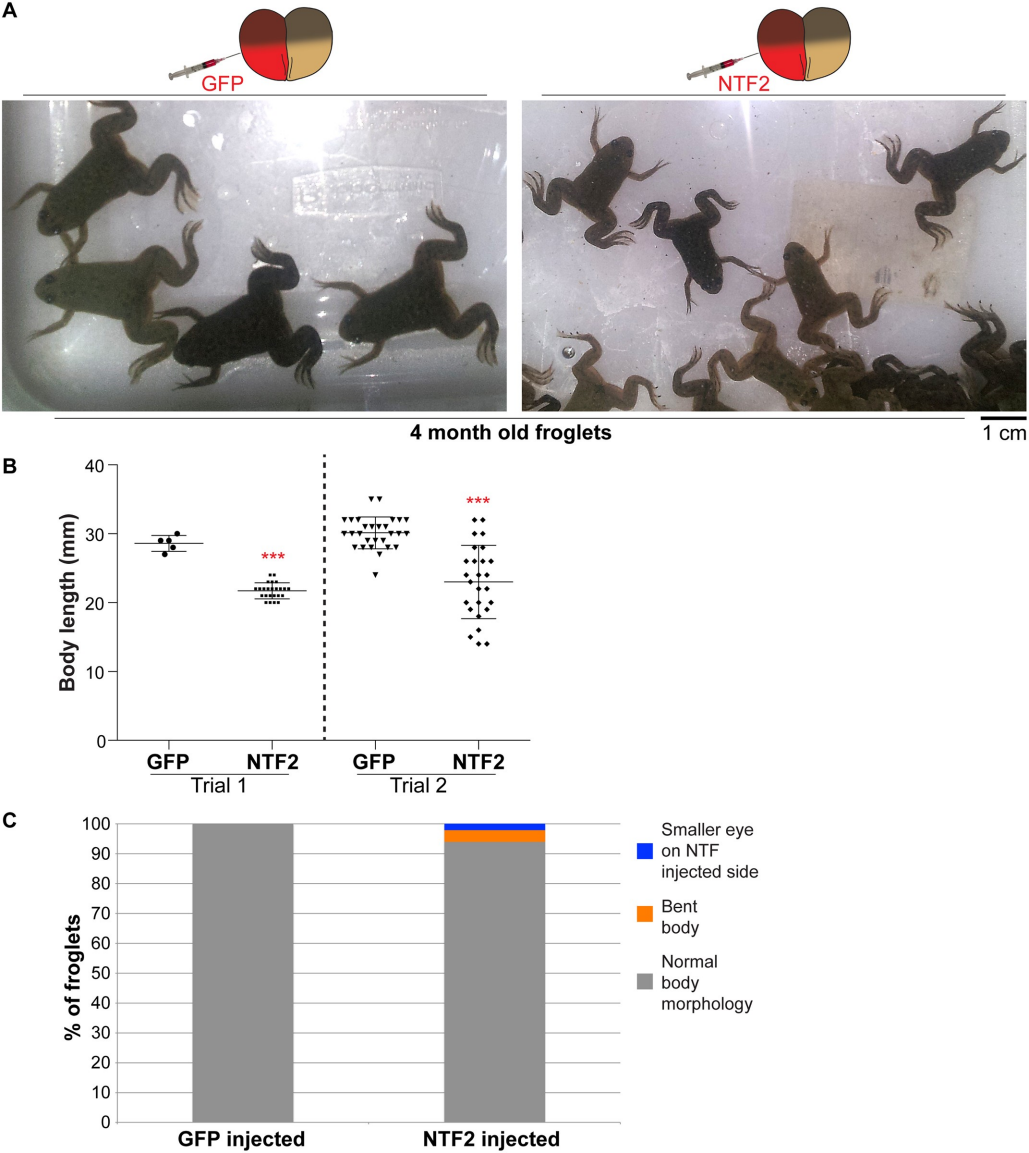
Differential levels of nuclear import factors in the two halves of an early embryo lead to smaller froglets. Two-cell embryos were microinjected as indicated and allowed to develop into 4-month-old froglets. Froglet numbers: trial 1 GFP n = 5, trial 1 NTF2 n = 24, trial 2 GFP n = 28, trial 2 NTF n = 26. **(A)** Representative froglets. **(B)** Quantification of froglet length is shown. Average body mass for froglets derived from NTF2-microinjected embryos was 59% ± 11% (average ± SD) of control froglets. Error bars represent SD. *** p<0.005. **(C)** Scoring of froglets with altered body morphology as indicated. We did not note any obvious differences in the timing of the onset of metamorphosis for GFP- and NTF2-injected animals.

We next asked if the bent neural plate phenotype was propagated later in development. More than 30% of NTF2-microinjected embryos exhibited a bent tadpole phenotype, again with the bend occurring toward the NTF2-microinjected side (Fig 3 and S4 and S5 Figs). We also observed more than 30% of tadpoles with a smaller eye on the NTF2-microinjected side, with 16% of embryos showing both the small eye and bent body phenotype (Fig 3 and S4 Fig). The small eye phenotype was exacerbated in embryos microinjected to maximize differences in nuclear import factor levels in the two halves of the embryo (Fig 3B). Reduced eye size may result from altered gene expression, due to increased NTF2 levels and/or altered nuclear size that might impact chromatin positioning [37–40]. Embryos microinjected at the one-cell stage did not develop into bent tadpoles (S5A Fig), similar to what was observed for neurula. Similar bent tadpoles were observed with frogs and embryos derived from two different frog colonies as well as for embryos microinjected with importin α/LB3 or importin α alone (Fig 3 and S5 Fig). Lastly, we allowed microinjected embryos to develop into 4-month-old froglets. NTF2-microinjected embryos gave rise to significantly smaller froglets, with a small proportion exhibiting defective body morphologies (Fig 4). We also observed that embryos microinjected with LB3 mRNA developed into larger froglets than NTF2-microinjected embryos (S6A Fig).

Taken together, we show that altering the levels of nuclear import factors in the early embryo leads to downstream effects on gastrulation, neurulation, and the development of tadpoles and froglets. Specifically, altering the levels of NTF2, importin α, and/or lamins in half of the embryo affects blastopore closure and leads to curved neural plates and bent tadpoles with different sized eyes. None of these effects were observed when one-cell embryos were microinjected (S3B and S5A Figs), although MBT timing was altered upon microinjection of one-cell stage embryos (S6C Fig) [24]. These results show how altering levels of nuclear import factors can, perhaps indirectly, affect function and size at the organismal level. Furthermore, given the defects we observed in neural plate and body morphologies, our findings may be relevant to a wide range of diseases associated with neural tube defects [41].

Why might transiently altering the levels of nuclear import factors in the early embryo lead to downstream effects on gastrulation, neurulation, and the development of tadpoles and froglets? Because nuclear import impinges on a variety of different cellular functions [14, 15], we cannot definitively state which altered function might be responsible for the observed effects on development. Altering levels of importin α and NTF2 could lead to differential nuclear import of cargos, affect importin α binding partners, or directly influence gene expression [42]. Our preferred model deals with nuclear size, as importin α/LB3 microinjection increased nuclear size while NTF2 microinjection decreased nuclear size (S1 Fig) [12, 13, 24]. We previously showed through a variety of different experimental manipulations that increasing nuclear size and the nuclear-to-cytoplasmic volume ratio leads to a premature MBT while decreasing nuclear size delays MBT onset [24, 25]. Relevant to the current study, importin α/LB3 microinjection accelerated MBT onset [24]. Consistent with these data, we find that NTF2 microinjection reduces zygotic gene expression, consistent with a delayed MBT (S6B and S6C Fig). Thus manipulating nuclear size using different approaches has consistent downstream developmental consequences, suggesting observed effects are due to nuclear size changes. We propose that altering nuclear size in the early embryo leads to changes in MBT timing that in turn impact cell size and number, subsequently disrupting later stages of development.

## Materials and methods

### Plasmids

Plasmids consisting of pCS2+ containing the coding sequences for human importin α2-E (pDL17), *X*. *tropicalis* GFP-LB3 (pDL19), and human NTF2 (pDL18) were described previously [12, 13]. For control injections, we used GFP mRNA expressed from pCS107-GFP3STOP or H2B-GFP mRNA expressed from CS2-H2BeGFP (gifts from John Wallingford, University of Texas at Austin). Of the three major importin α isoforms in *Xenopus*, we chose importin α2 because previous work identified it as an interspecies and developmental regulator of nuclear size [12, 24, 43]. Human and *X*. *laevis* importin α2 are 76% identical and 88% similar, and both affect nuclear size in *Xenopus* extracts and embryos [12]. Human and *X*. *laevis* NTF2 are 87% identical and 92% similar.

### *Xenopus laevis* embryos and microinjections

*X*. *laevis* embryos were obtained by in vitro fertilization of freshly laid *X*. *laevis* eggs with crushed *X*. *laevis* testes [44]. Only batches with greater than 90% fertilization efficiency were used. Twenty minutes after fertilization, embryos were de-jellied in 2.5% cysteine pH 7.8 dissolved in 1/3x MMR (20x MMR = 2 mM EDTA, 2 M NaCl, 40 mM KCl, 20 mM MgCl_2_, 40 mM CaCl_2_, 100 mM HEPES pH 7.8). Embryos were staged according to [45]. All *Xenopus* procedures and studies were conducted in compliance with the US Department of Health and Human Services Guide for the Care and Use of Laboratory Animals. Protocols were approved by the University of Wyoming Institutional Animal Care and Use Committee (Assurance # A-3216-01).

Following linearization of pCS107-GFP3STOP, CS2-H2BeGFP, pDL17, pDL18, and pDL19, mRNA was expressed from the SP6 promoter using the mMessage mMachine kit (Ambion). Embryos at the one-cell or two-cell stage were transferred to 1/3 MMR plus 2.5% Ficoll and microinjected with 10 nL volumes using a PicoSpritzer III (Parker). Different amounts of mRNA were injected by varying the concentration of the mRNA stock solution. Unless otherwise indicated, the following mRNA amounts were used for each microinjection: 250 pg GFP, 100 pg H2B-GFP, 175 pg NTF2, 250 pg importin α, 250 pg GFP-LB3. This amount of NTF2 mRNA maximally decreases nuclear size [13], while these amounts of importin α and GFP-LB3 mRNA maximally increase nuclear size [24]. After 45 minutes, the buffer was changed to 1/3x MMR and embryos were allowed to develop to desired stages. Tadpoles were grown in 1/3x MMR in small tanks with water filtration at room temperature. During metamorphosis, froglets were grown in the same water as adults at room temperature. Tadpoles and froglets were fed tadpole frog brittle (Nasco SA05964) and post-metamorphic frog brittle (Nasco SB29027), respectively. GFP-LB3 is primarily nuclear (S4D Fig), while importin α and NTF2 can be found in both the nucleus and cytoplasm [1, 2, 6–10, 12, 13, 16, 46, 47].

In most experiments, one blastomere of a two-cell embryo was co-microinjected with mRNA and 50 ng of a fluorescently labeled dextran that served as a marker for the injected half. Dextrans used were lysine-fixable tetramethylrhodamine-labeled dextran, 70,000 MW (ThermoFisher, D1818) or lysine-fixable fluorescein-labeled dextran, 70,000 MW (ThermoFisher, D1822). For control experiments, mRNA expressing GFP or H2B-GFP was used; few to none of these embryos exhibited altered developmental phenotypes indicating that potential effects of microinjected mRNA on translation rates of endogenous transcripts were negligible. Visualization of fluorescently-labeled dextrans indicated that microinjected molecules were fairly uniformly distributed throughout the injected side of the embryo, confirmed by expression of H2B-GFP (S3 Video and S3A Fig) and consistent with our previous studies [24, 25].

Whole-mount fluorescence immunocytochemistry was performed following a published protocol [48]. The primary antibody was anti-tubulin-β 2B (TUBB2B) used at a 1:50 dilution (BioLegend #909101). The secondary antibody was goat anti-mouse IgG (H+L) Alexa Fluor 488 used at a 1:250 dilution (Life Technologies #A28175). Prior to imaging, immunostained embryos were cleared in 2:1 benzyl benzoate/benzyl alcohol [49]. Whole-mount in situ hybridizations and qPCR were performed as previously described [24, 25].

### Microscopy and image quantification

For S1 Fig, microinjected embryos at stage 11 were transferred to 1/3x MMR containing 10 μg/ml Hoechst. Subsequently, embryos were squashed between a glass coverslip and slide for imaging. Nuclei were visualized with an Olympus BX51 fluorescence microscope using an Olympus UPLFLN 20x (N.A. 0.50, air) objective. Images were acquired with a QIClick Digital CCD Camera, Mono, 12-bit (model QIClick-F-M-12) at room temperature using Olympus cellSens software. Nuclear cross-sectional areas were quantified from original thresholded images using cellSens Dimension imaging software (Olympus).

Brightfield and fluorescence imaging of eggs, embryos, and tadpoles was performed with an Olympus SZX16 research fluorescence stereomicroscope, equipped with Olympus DP72 camera, 11.5x zoom microscope body, and SDFPLAPO1XPF objective. Brightfield time-lapse imaging of embryos was performed at room temperature, and images were acquired every 5 minutes. Discontinuous light was used to illuminate embryos, controlled with a digital adjustable cycle timer (CT-1 Short Cycle Timer, Innovative Grower Corp). Swimming tadpoles were anesthetized in 1/3x MMR containing 0.05% benzocaine prior to imaging. Blastopore areas were quantified from original thresholded images using cellSens Dimension imaging software (Olympus). Eye areas and body angles in swimming tadpoles were measured from original images using cellSens Dimension imaging software measurement tools (Olympus). Froglets were anesthetized in 0.05% benzocaine to measure body mass and length. Froglets and frogs were imaged in plastic containers using a cell phone camera.

Where indicated, confocal imaging was performed on a spinning-disk confocal microscope based on an Olympus IX71 microscope stand equipped with a five line LMM5 laser launch (Spectral Applied Research) and Yokogawa CSU-X1 spinning-disk head. Confocal images were acquired with an EM-CCD camera (ImagEM, Hamamatsu). Z-axis focus was controlled using a piezo Pi-Foc (Physik Instrumentes), and multiposition imaging was achieved using a motorized Ludl stage. An Olympus UPLSAPO 20xO/0.85na objective was used. Image acquisition and all system components were controlled using Metamorph software.

### Statistics

Averaging and statistical analysis were performed for independently repeated experiments. Two-tailed Student’s t tests assuming equal variances were performed in Excel (Microsoft) to evaluate statistical significance. The p values, sample sizes, and error bars are given in the figure legends.

## Supporting information

S1 Fig Effects of NTF2 and importin α/lamin B3 microinjection on nuclear size. We previously demonstrated that microinjection of *X*. *laevis* single-cell embryos with mRNA encoding NTF2 alone or importin α + GFP-lamin B3 resulted in altered nuclear size in stage 8 embryos. After testing a range of mRNA amounts, we determined that 350 pg of NTF2 mRNA maximally decreased nuclear size [13] while co-microinjection of 500 pg each of importin α and GFP-LB3 mRNA maximally increased nuclear size [12, 24]. To confirm that these nuclear size effects were detectable in gastrula stage embryos, we microinjected half of these mRNA amounts into one blastomere of two-cell stage embryos, allowed the embryos to develop to stage 11, and quantified nuclear sizes. **(A)** Two-cell embryos were microinjected as indicated with 250 pg GFP mRNA, 175 pg NTF mRNA, or 250 pg importin α mRNA + 250 pg GFP-LB3 mRNA and allowed to develop to 11 hpf gastrula. Embryos were stained with Hoechst. Representative images are shown. **(B)** Nuclei in dextran-injected cells on the embryo surface were imaged and nuclear cross-sectional areas were quantified. For each condition, 10–20 embryos were analyzed and 68–72 nuclei were quantified. Error bars represent SD. *** p<0.005. Compared to cells that received GFP mRNA (250 pg), NTF2 mRNA microinjection (175 pg) decreased nuclear area by 31% and importin α + GFP-LB3 mRNA co-microinjection (250 pg each) increased nuclear area by 38%. These amounts of mRNA were therefore used throughout the rest of this study. **(C)** One-cell embryos were microinjected with the indicated amounts of importin α mRNA and allowed to develop to late stage 8. Embryo extracts were prepared and analyzed by western blot as previously described [12]. One representative importin α western blot is shown. Note that ectopically expressed human importin α runs faster than endogenous *X*. *laevis* importin α. Based on two experiments, 500 pg importin α mRNA increased the importin α level by 61% ± 2% (average ± SD), relative to the endogenous level. Given an endogenous total importin α concentration of 5 μM in stage 8 embryos [12, 43], the ectopic importin α concentration in microinjected embryos was 3 μM. Previous work quantified the endogenous NTF2 concentration at 0.7 μM in stage 8 embryos [12]. When single-cell embryos were microinjected with 350 pg of NTF2 mRNA, the NTF2 concentration in late stage 8 embryos was increased by 1.2 μM [13]. Previous work quantified the endogenous LB3 concentration at 25 nM in stage 12 embryos [12]. When single-cell embryos were microinjected with 500 pg of LB3 mRNA, the LB3 concentration in stage 11–12 embryos was increased by 170 nM [16]. Amounts of microinjected mRNA were empirically selected to maximize effects on nuclear size [12, 13, 24].
(TIF)

S2 Fig Differential levels of nuclear import factors in the two halves of an early embryo lead to asymmetric neural plate closure and cell size differences. **(A)** Two-cell embryos were microinjected as indicated and allowed to develop to 20 hpf neurula. Representative images are shown from S1 Video. Note that the dextran images were acquired at the beginning of the time-lapse while the brightfield images were selected later in the time-lapse to highlight asymmetric neural plate closure. For this reason, the brightfield and dextran images do not perfectly align, with the dextran image simply showing the side of the embryo that was microinjected. For the NTF2 microinjection image, a still frame was selected that shows delayed neural plate closure on the microinjected side, however bending of the neural plate toward the microinjected side does not become apparent until later in the time-lapse (see S1B Video). **(B)** Two-cell embryos were microinjected as indicated and allowed to develop to stage 8. Representative confocal embryo surface images are shown. The cross-sectional areas of surface exposed cells were quantified for both the uninjected and injected sides of the embryo. Average cell areas were normalized to the uninjected controls. For importin α, 25 and 13 cells were quantified on the uninjected and injected sides, respectively. For NTF2, 11 and 17 cells were quantified on the uninjected and injected sides, respectively. Error bars represent SD. *** p<0.005. **(C)** Microinjections were performed as indicated and embryos were allowed to develop to 24 hpf. Representative confocal embryo surface images are shown.
(TIF)

S3 Fig Differential levels of nuclear import factors in the two halves of an early embryo lead to neural plate curvature. **(A)** Two-cell embryos were microinjected as indicated and allowed to develop to 22 hpf neurula. Representative images are shown. **(B)** One blastomere of a two-cell embryo was microinjected with importin α + GFP-LB3 as indicated in the first column. One-cell embryos were microinjected with NTF2 or importin α + GFP-LB3 as indicated in the second and third columns, respectively. Embryos were allowed to develop to 22 hpf neurula. Representative images are shown. Neurula were scored as having normal or curved neural plates by drawing a line through the middle of the embryo. Embryo numbers: n = 19 for GFP, n = 22 for imp α + GFP-LB3 injected into one cell at 2-cell stage, n = 10 for NTF2 injected at the 1-cell stage, n = 39 for imp α + GFP-LB3 injected at the 1-cell stage. The GFP microinjection quantification is the same as shown in Fig 2B. Data presented in Fig 2 and S3A and S3B Fig were generated from two different frog colonies. **(C)** Two-cell embryos were microinjected as indicated and allowed to develop to 22 hpf neurula. Whole-mount fluorescence immunocytochemistry was performed using an anti-tubulin-β 2B (TUBB2B) antibody. Representative neural plates are shown. TUBB2B staining intensity was measured on the two sides of the neural plate, and the intensity on the injected side was divided by the intensity on the uninjected side. Embryo numbers: n = 12 for GFP, n = 14 for NTF2, n = 10 for imp α + GFP-LB3. Error bars represent SD. NS not significant.
(TIF)

S4 Fig Quantifying defects in tadpole body morphology. **(A)** Two-cell embryos were microinjected as indicated and allowed to develop into 9 dpf swimming tadpoles. Representative images are shown. Eye areas were measured from brightfield images, as shown in red text. Body axis bend angles were measured from brightfield images, as shown in green text. Note that the bent tadpole microinjected with NTF2 is the same one shown in Fig 3A. **(B)** For each tadpole, the area of the eye on the injected side was divided by the area of the eye on the uninjected side to obtain the eye size ratio. Average ratios are plotted for 3–7 tadpoles per condition. These ratios were compared to the GFP injected controls to identify tadpoles with small eyes on the injected side having eye size ratios less than 1. Error bars represent SD. ** p<0.01, * p<0.05. **(C)** Average body axis angles are plotted for 4–5 tadpoles per condition. Error bars represent SD. *** p<0.005, * p<0.05. **(D)** One blastomere of a two-cell embryo was microinjected with GFP-LB3 mRNA. The embryo was allowed to develop into a tadpole. After staining with Hoechst, nuclei in the tadpole tail were visualized as indicated. GFP-LB3 expression persists in the tadpole. Nuclei expressing GFP-LB3 are larger than nuclei in non-expressing neighboring cells, visible only by Hoechst-staining in the upper panel. These results are consistent with studies showing that protein expression from microinjected mRNA can persist up to several days [50, 51].
(TIF)

S5 Fig Differential levels of nuclear import factors in the two halves of an early embryo lead to bent tadpoles. **(A)** Column 1: uninjected embryo. Column 2: One-cell embryo was microinjected with NTF2. Column 3: One blastomere of a two-cell embryo was microinjected with NTF2. Column 4: One-cell embryo was microinjected with importin α. Column 5: One blastomere of a two-cell embryo was microinjected with importin α. Embryos were allowed to develop into 5–6 dpf swimming tadpoles. Representative images are shown. Double-headed arrows indicate bent bodies. **(B)** Two-cell embryos were microinjected as indicated and allowed to develop into 9 dpf swimming tadpoles. Tadpoles were scored as indicated by measuring body axis angle. Embryo numbers: n = 10 for GFP, n = 10 for imp α + GFP-LB3. The GFP microinjection quantification is the same as shown in Fig 3B. Data presented in Fig 3 and S5 Fig were generated from two different frog colonies.
(TIF)

S6 Fig Lamin B3 affects froglet size and NTF2 affects the timing of zygotic gene expression. **(A)** At the two-cell stage one blastomere was microinjected with the indicated mRNA, and embryos were allowed to develop into froglets. Froglet body mass and length were quantified. Froglet numbers: n = 3 for lamin B3 and n = 3 for NTF2. **(B)** One blastomere of a two-cell embryo was co-microinjected with rhodamine-labeled dextran and NTF2 mRNA. Post-MBT embryos were subjected to in situ hybridization to detect the GS17 transcript. The top panels are bright-field images of embryos stained for GS17 (purple). The bottom panels are the corresponding rhodamine fluorescence images indicating cells in the embryo that received the NTF2 mRNA. Representative embryos are shown. **(C)** One-cell embryos were microinjected with GFP or NTF2 mRNA and allowed to develop to post-MBT (7.5 hpf). Total RNA was isolated from 12 embryos for each condition and converted to cDNA. Expression levels of three zygotic genes (xnr5-13, xnr3, and bix1.1) were determined by qPCR, normalized to ODC. Gene-expression levels are plotted in arbitrary units (AU) relative to GFP mRNA-injected control embryos. The means from 2 independent experiments are shown. Error bars represent SD. *** p<0.005, * p<0.05, NS not significant.
(TIF)

S1 Video Neural plate closure. Two-cell embryos were microinjected and allowed to develop to 20 hpf neurula at 22°C. Brightfield imaging was performed at 5 minute intervals. Note that the embryos shown in this video are from different batches of eggs that were microinjected and imaged on different days, therefore developmental timing cannot be compared between these different embryos. **(A)** A two-cell embryo was microinjected in the left side with GFP mRNA and, the total length of the time-lapse is 4 hours. **(B)** A two-cell embryo was microinjected in the left side with NTF2 mRNA, and the total length of the time-lapse is 4 hours. **(C)** A two-cell embryo was microinjected in the left side with importin α + GFP-LB3 mRNA, and the total length of the time-lapse is 5.5 hours. **(D)** A two-cell embryo was microinjected with importin α + GFP-LB3 mRNA in the left side and with NTF2 mRNA in the right side, and the total length of the time-lapse is 9 hours.
(AVI)

S2 Video Importin α + GFP-LB3 versus NTF2 microinjection neurula. A two-cell embryo was microinjected with importin α + GFP-LB3 mRNA in the left side and with NTF2 mRNA in the right side. The embryo was allowed to develop to a 22 hpf neurula at 22°C. Brightfield imaging was performed at 5 minute intervals. The total length of the time-lapse is 3 hours.
(MOV)

S3 Video NTF2 microinjection neurula. For the top two embryos, one blastomere of a two-cell embryo was co-microinjected with red dextran and H2B-GFP mRNA. For the bottom two embryos, one blastomere of a two-cell embryo was co-microinjected with NTF2 mRNA, red dextran, and H2B-GFP mRNA. Embryos were allowed to develop to 22 hpf neurula at 22°C. Brightfield imaging was performed at 5 minute intervals. Red and green fluorescence images at the beginning and end of the movie show the sides of the embryos that were microinjected. The total length of the time-lapse is 4.5 hours.
(MOV)

## Abbreviations

dpf: days post fertilization
hpf: hours post fertilization
imp α: importin α
LB3: lamin B3
MBT: midblastula transition
NE: nuclear envelope
NLS: nuclear localization signal
NPC: nuclear pore complex
NTF2: nuclear transport factor 2

## References

[1] Dittmer TA, Misteli T. The lamin protein family. Genome Biol. 2011;12(5):222 10.1186/gb-2011-12-5-222 21639948 PMC3219962

[2] Wilson KL, Berk JM. The nuclear envelope at a glance. J Cell Sci. 2010;123(Pt 12):1973–8. 10.1242/jcs.019042 20519579 PMC2880010

[3] Rothballer A, Kutay U. SnapShot: the nuclear envelope II. Cell. 2012;150(5):1084–e1. 10.1016/j.cell.2012.08.003 22939630

[4] Rothballer A, Kutay U. SnapShot: The nuclear envelope I. Cell. 2012;150(4):868–e1. 10.1016/j.cell.2012.07.024 22901815

[5] Misteli T, Spector DL. The Nucleus. Cold Spring Harbor, New York: Cold Spring Harbor Laboratory Press; 2011 517 p.

[6] Madrid AS, Weis K. Nuclear transport is becoming crystal clear. Chromosoma. 2006;115(2):98–109. 10.1007/s00412-005-0043-3 16421734

[7] Stewart M. Molecular mechanism of the nuclear protein import cycle. Nat Rev Mol Cell Biol. 2007;8(3):195–208. 10.1038/nrm2114 17287812

[8] Fried H, Kutay U. Nucleocytoplasmic transport: taking an inventory. Cell Mol Life Sci. 2003;60(8):1659–88. 10.1007/s00018-003-3070-3 14504656 PMC11138860

[9] Dickmanns A, Kehlenbach RH, Fahrenkrog B. Nuclear Pore Complexes and Nucleocytoplasmic Transport: From Structure to Function to Disease. Int Rev Cell Mol Biol. 2015;320:171–233. 10.1016/bs.ircmb.2015.07.010 26614874

[10] Hutten S, Kehlenbach RH. CRM1-mediated nuclear export: to the pore and beyond. Trends Cell Biol. 2007;17(4):193–201. 10.1016/j.tcb.2007.02.003 17317185

[11] Feldherr C, Akin D, Moore MS. The nuclear import factor p10 regulates the functional size of the nuclear pore complex during oogenesis. J Cell Sci. 1998;111 (Pt 13):1889–96. 9625751 10.1242/jcs.111.13.1889

[12] Levy DL, Heald R. Nuclear size is regulated by importin alpha and Ntf2 in Xenopus. Cell. 2010;143(2):288–98. 10.1016/j.cell.2010.09.012 20946986 PMC2966892

[13] Vukovic LD, Jevtic P, Zhang Z, Stohr BA, Levy DL. Nuclear size is sensitive to NTF2 protein levels in a manner dependent on Ran binding. J Cell Sci. 2016;129(6):1115–27. 10.1242/jcs.181263 26823604 PMC4813295

[14] Macara IG. Transport into and out of the nucleus. Microbiol Mol Biol Rev. 2001;65(4):570–94, table of contents. 10.1128/MMBR.65.4.570-594.2001 11729264 PMC99041

[15] Mackmull MT, Klaus B, Heinze I, Chokkalingam M, Beyer A, Russell RB, et al Landscape of nuclear transport receptor cargo specificity. Mol Syst Biol. 2017;13(12):962 10.15252/msb.20177608 29254951 PMC5740495

[16] Jevtic P, Edens LJ, Li X, Nguyen T, Chen P, Levy DL. Concentration-dependent Effects of Nuclear Lamins on Nuclear Size in Xenopus and Mammalian Cells. J Biol Chem. 2015;290(46):27557–71. 10.1074/jbc.M115.673798 26429910 PMC4646008

[17] Newport J, Kirschner M. A major developmental transition in early Xenopus embryos: I. characterization and timing of cellular changes at the midblastula stage. Cell. 1982;30(3):675–86. 6183003 10.1016/0092-8674(82)90272-0

[18] Newport J, Kirschner M. A major developmental transition in early Xenopus embryos: II. Control of the onset of transcription. Cell. 1982;30(3):687–96. 7139712 10.1016/0092-8674(82)90273-2

[19] Newport JW, Kirschner MW. Regulation of the cell cycle during early Xenopus development. Cell. 1984;37(3):731–42. 6378387 10.1016/0092-8674(84)90409-4

[20] Collart C, Owens ND, Bhaw-Rosun L, Cooper B, De Domenico E, Patrushev I, et al High-resolution analysis of gene activity during the Xenopus mid-blastula transition. Development. 2014;141(9):1927–39. 10.1242/dev.102012 24757007 PMC3994770

[21] Clute P, Masui Y. Regulation of the appearance of division asynchrony and microtubule-dependent chromosome cycles in Xenopus laevis embryos. Dev Biol. 1995;171(2):273–85. 10.1006/dbio.1995.1280 7556912

[22] Kobayakawa Y, Kubota HY. Temporal pattern of cleavage and the onset of gastrulation in amphibian embryos developed from eggs with the reduced cytoplasm. J Embryol Exp Morphol. 1981;62:83–94. 7276823

[23] Clute P, Masui Y. Microtubule dependence of chromosome cycles in Xenopus laevis blastomeres under the influence of a DNA synthesis inhibitor, aphidicolin. Dev Biol. 1997;185(1):1–13. 10.1006/dbio.1997.8540 9169045

[24] Jevtic P, Levy DL. Nuclear size scaling during Xenopus early development contributes to midblastula transition timing. Curr Biol. 2015;25(1):45–52. 10.1016/j.cub.2014.10.051 25484296 PMC4286459

[25] Jevtic P, Levy DL. Both Nuclear Size and DNA Amount Contribute to Midblastula Transition Timing in Xenopus laevis. Sci Rep. 2017;7(1):7908 10.1038/s41598-017-08243-z 28801588 PMC5554259

[26] Gorlich D, Seewald MJ, Ribbeck K. Characterization of Ran-driven cargo transport and the RanGTPase system by kinetic measurements and computer simulation. Embo J. 2003;22(5):1088–100. 10.1093/emboj/cdg113 12606574 PMC150346

[27] Riddick G, Macara IG. A systems analysis of importin-{alpha}-{beta} mediated nuclear protein import. J Cell Biol. 2005;168(7):1027–38. 10.1083/jcb.200409024 15795315 PMC2171841

[28] Riddick G, Macara IG. The adapter importin-alpha provides flexible control of nuclear import at the expense of efficiency. Mol Syst Biol. 2007;3:118 10.1038/msb4100160 17551513 PMC1911202

[29] Smith AE, Slepchenko BM, Schaff JC, Loew LM, Macara IG. Systems analysis of Ran transport. Science. 2002;295(5554):488–91. 10.1126/science.1064732 11799242

[30] Ma Y, Zhang P, Wang F, Yang J, Yang Z, Qin H. The relationship between early embryo development and tumourigenesis. J Cell Mol Med. 2010;14(12):2697–701. 10.1111/j.1582-4934.2010.01191.x 21029369 PMC3822720

[31] Aiello NM, Stanger BZ. Echoes of the embryo: using the developmental biology toolkit to study cancer. Dis Model Mech. 2016;9(2):105–14. 10.1242/dmm.023184 26839398 PMC4770149

[32] Jevtic P, Levy DL. Mechanisms of nuclear size regulation in model systems and cancer. Adv Exp Med Biol. 2014;773:537–69. 10.1007/978-1-4899-8032-8_25 24563365

[33] Jevtic P, Levy DL. Elucidating nuclear size control in the Xenopus model system. Veterinarski Glasnik. 2018;72(1):1–13. 10.2298/VETGL170731012J 30474651 PMC6242335

[34] Amodeo AA, Jukam D, Straight AF, Skotheim JM. Histone titration against the genome sets the DNA-to-cytoplasm threshold for the Xenopus midblastula transition. Proc Natl Acad Sci U S A. 2015;112(10):E1086–95. 10.1073/pnas.1413990112 25713373 PMC4364222

[35] Collart C, Allen GE, Bradshaw CR, Smith JC, Zegerman P. Titration of four replication factors is essential for the Xenopus laevis midblastula transition. Science. 2013;341(6148):893–6. 10.1126/science.1241530 23907533 PMC3898016

[36] Wang P, Hayden S, Masui Y. Transition of the blastomere cell cycle from cell size-independent to size-dependent control at the midblastula stage in Xenopus laevis. J Exp Zool. 2000;287(2):128–44. 10900432 10.1002/1097-010x(20000701)287:2<128::aid-jez3>3.0.co;2-g

[37] Bugner V, Aurhammer T, Kuhl M. Xenopus laevis insulin receptor substrate IRS-1 is important for eye development. Dev Dyn. 2011;240(7):1705–15. 10.1002/dvdy.22659 21574211

[38] Zuber ME, Perron M, Philpott A, Bang A, Harris WA. Giant eyes in Xenopus laevis by overexpression of XOptx2. Cell. 1999;98(3):341–52. 10458609 10.1016/s0092-8674(00)81963-7

[39] Rasmussen JT, Deardorff MA, Tan C, Rao MS, Klein PS, Vetter ML. Regulation of eye development by frizzled signaling in Xenopus. Proc Natl Acad Sci U S A. 2001;98(7):3861–6. 10.1073/pnas.071586298 11274406 PMC31143

[40] Cizelsky W, Hempel A, Metzig M, Tao S, Hollemann T, Kuhl M, et al sox4 and sox11 function during Xenopus laevis eye development. PLoS One. 2013;8(7):e69372 10.1371/journal.pone.0069372 23874955 PMC3715537

[41] Copp AJ, Greene ND. Neural tube defects—disorders of neurulation and related embryonic processes. Wiley Interdiscip Rev Dev Biol. 2013;2(2):213–27. 10.1002/wdev.71 24009034 PMC4023228

[42] Briggs JA, Weinreb C, Wagner DE, Megason S, Peshkin L, Kirschner MW, et al The dynamics of gene expression in vertebrate embryogenesis at single-cell resolution. Science. 2018;360(6392). 10.1126/science.aar5780 PMC6038144 29700227

[43] Wilbur JD, Heald R. Mitotic spindle scaling during Xenopus development by kif2a and importin alpha. eLife. 2013;2:e00290 10.7554/eLife.00290 23425906 PMC3576809

[44] Sive HL, Grainger RM, Harland RM. Early development of Xenopus laevis: a laboratory manual. Cold Spring Harbor, N.Y: Cold Spring Harbor Laboratory Press; 2000 ix, 338 p. p.

[45] Nieuwkoop PD, Faber J. Normal Table of Xenopus laevis (Daudin). 2nd ed Amsterdam: North-Holland Publishing Company; 1967.

[46] Edens LJ, Levy DL. cPKC regulates interphase nuclear size during Xenopus development. J Cell Biol. 2014;206(4):473–83. 10.1083/jcb.201406004 25135933 PMC4137061

[47] Edens LJ, Dilsaver MR, Levy DL. PKC-mediated phosphorylation of nuclear lamins at a single serine residue regulates interphase nuclear size in Xenopus and mammalian cells. Mol Biol Cell. 2017;28(10):1389–99. 10.1091/mbc.E16-11-0786 28356420 PMC5426852

[48] Lee C, Kieserman E, Gray RS, Park TJ, Wallingford J. Whole-mount fluorescence immunocytochemistry on Xenopus embryos. CSH Protoc. 2008;2008:pdb prot4957. 10.1101/pdb.prot4957 21356778

[49] Wallingford JB. Preparation of fixed Xenopus embryos for confocal imaging. Cold Spring Harb Protoc. 2010;2010(5):pdb prot5426. 10.1101/pdb.prot5426 20439413

[50] Mimoto MS, Christian JL. Manipulation of gene function in Xenopus laevis. Methods Mol Biol. 2011;770:55–75. 10.1007/978-1-61779-210-6_3 21805261 PMC3911881

[51] Souopgui J, Rust B, Vanhomwegen J, Heasman J, Henningfeld KA, Bellefroid E, et al The RNA-binding protein XSeb4R: a positive regulator of VegT mRNA stability and translation that is required for germ layer formation in Xenopus. Genes Dev. 2008;22(17):2347–52. 10.1101/gad.479808 18765788 PMC2532925

