# Supplementary figures and images for: Altering the levels of nuclear import factors in early Xenopus laevis embryos affects later development
Source: PLoS One. 2019 Apr 22;14(4):e0215740. doi: 10.1371/journal.pone.0215740 (PMC6476522; doi:10.1371/journal.pone.0215740)

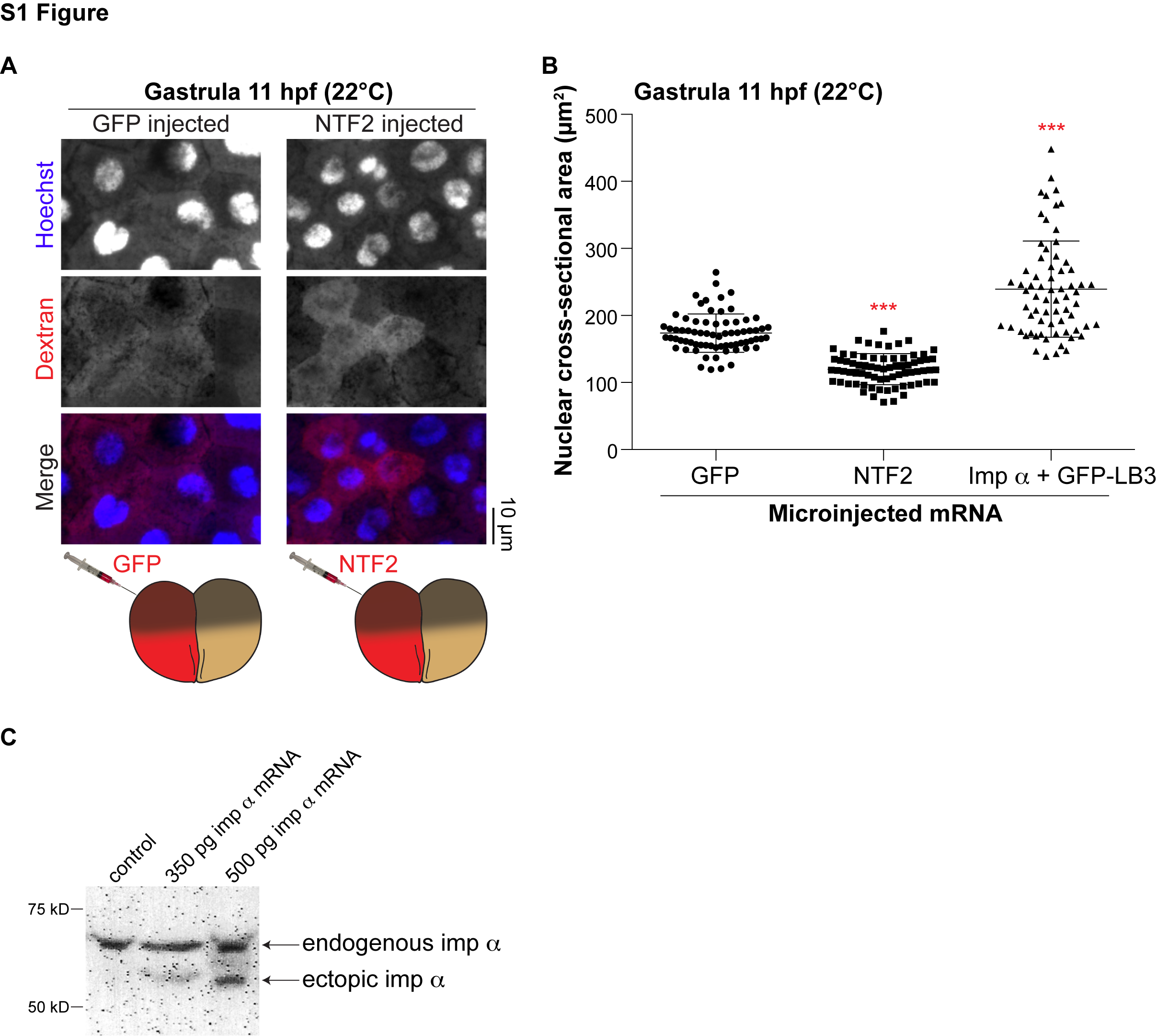

Supplement: S1 Fig — We previously demonstrated that microinjection of X. laevis single-cell embryos with mRNA encoding NTF2 alone or importin α + GFP-lamin B3 resulted in altered nuclear size in stage 8 embryos. After testing a range of mRNA amounts, we determined that 350 pg of NTF2 mRNA maximally decreased nuclear size [13] while co-microinjection of 500 pg each of importin α and GFP-LB3 mRNA maximally increased nuclear size [12, 24]. To confirm that these nuclear size effects were detectable in gastrula stage embryos, we microinjected half of these mRNA amounts into one blastomere of two-cell stage embryos, allowed the embryos to develop to stage 11, and quantified nuclear sizes. (A) Two-cell embryos were microinjected as indicated with 250 pg GFP mRNA, 175 pg NTF mRNA, or 250 pg importin α mRNA + 250 pg GFP-LB3 mRNA and allowed to develop to 11 hpf gastrula. Embryos were stained with Hoechst. Representative images are shown. (B) Nuclei in dextran-injected cells on the embryo surface were imaged and nuclear cross-sectional areas were quantified. For each condition, 10–20 embryos were analyzed and 68–72 nuclei were quantified. Error bars represent SD. *** p<0.005. Compared to cells that received GFP mRNA (250 pg), NTF2 mRNA microinjection (175 pg) decreased nuclear area by 31% and importin α + GFP-LB3 mRNA co-microinjection (250 pg each) increased nuclear area by 38%. These amounts of mRNA were therefore used throughout the rest of this study. (C) One-cell embryos were microinjected with the indicated amounts of importin α mRNA and allowed to develop to late stage 8. Embryo extracts were prepared and analyzed by western blot as previously described [12]. One representative importin α western blot is shown. Note that ectopically expressed human importin α runs faster than endogenous X. laevis importin α. Based on two experiments, 500 pg importin α mRNA increased the importin α level by 61% ± 2% (average ± SD), relative to the endogenous level. Given an endogenous total importin α conc [file pone.0215740.s001.tif]

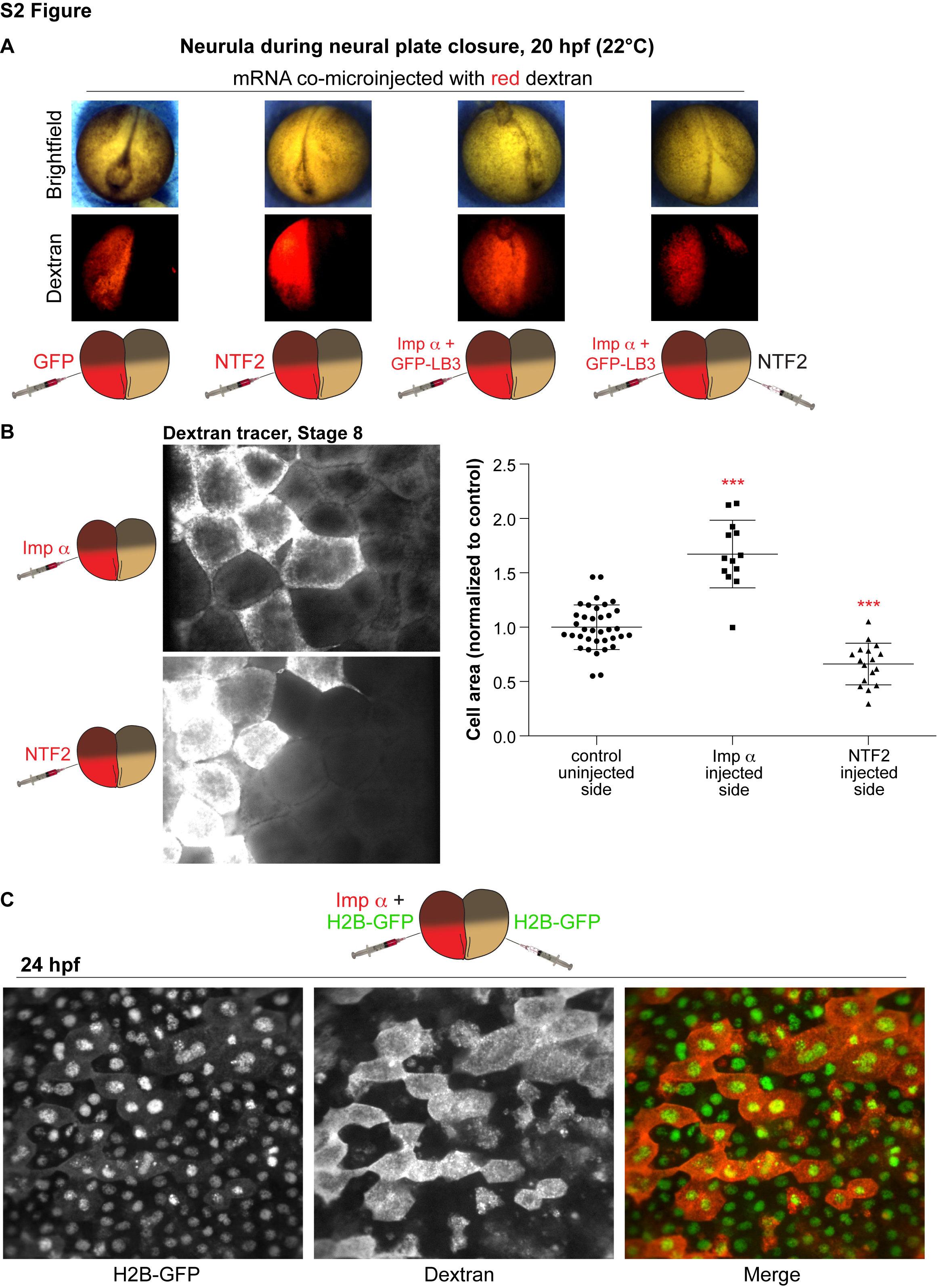

Supplement: S2 Fig — (A) Two-cell embryos were microinjected as indicated and allowed to develop to 20 hpf neurula. Representative images are shown from S1 Video. Note that the dextran images were acquired at the beginning of the time-lapse while the brightfield images were selected later in the time-lapse to highlight asymmetric neural plate closure. For this reason, the brightfield and dextran images do not perfectly align, with the dextran image simply showing the side of the embryo that was microinjected. For the NTF2 microinjection image, a still frame was selected that shows delayed neural plate closure on the microinjected side, however bending of the neural plate toward the microinjected side does not become apparent until later in the time-lapse (see S1B Video). (B) Two-cell embryos were microinjected as indicated and allowed to develop to stage 8. Representative confocal embryo surface images are shown. The cross-sectional areas of surface exposed cells were quantified for both the uninjected and injected sides of the embryo. Average cell areas were normalized to the uninjected controls. For importin α, 25 and 13 cells were quantified on the uninjected and injected sides, respectively. For NTF2, 11 and 17 cells were quantified on the uninjected and injected sides, respectively. Error bars represent SD. *** p<0.005. (C) Microinjections were performed as indicated and embryos were allowed to develop to 24 hpf. Representative confocal embryo surface images are shown. (TIF) [file pone.0215740.s002.tif]

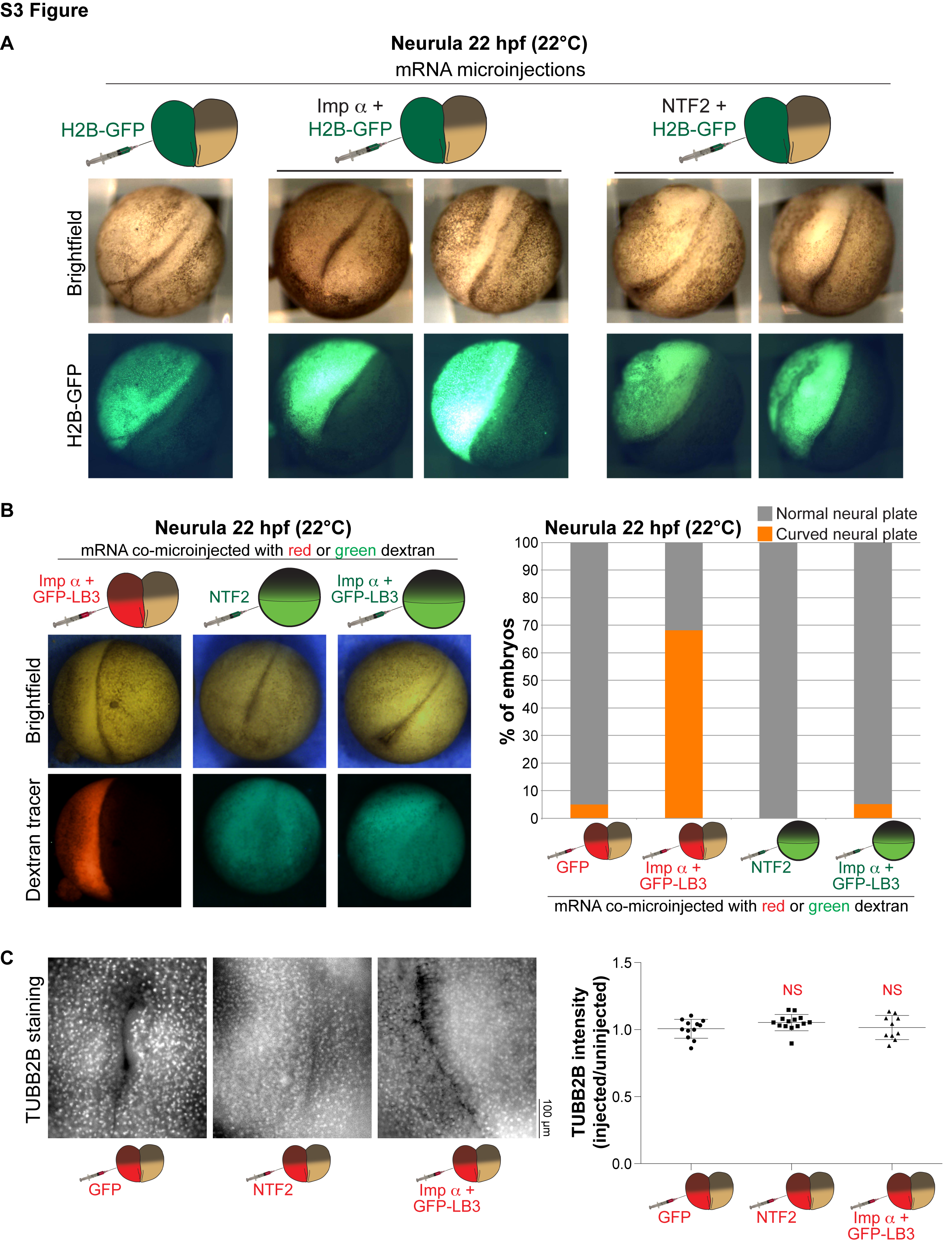

Supplement: S3 Fig — (A) Two-cell embryos were microinjected as indicated and allowed to develop to 22 hpf neurula. Representative images are shown. (B) One blastomere of a two-cell embryo was microinjected with importin α + GFP-LB3 as indicated in the first column. One-cell embryos were microinjected with NTF2 or importin α + GFP-LB3 as indicated in the second and third columns, respectively. Embryos were allowed to develop to 22 hpf neurula. Representative images are shown. Neurula were scored as having normal or curved neural plates by drawing a line through the middle of the embryo. Embryo numbers: n = 19 for GFP, n = 22 for imp α + GFP-LB3 injected into one cell at 2-cell stage, n = 10 for NTF2 injected at the 1-cell stage, n = 39 for imp α + GFP-LB3 injected at the 1-cell stage. The GFP microinjection quantification is the same as shown in Fig 2B. Data presented in Fig 2 and S3A and S3B Fig were generated from two different frog colonies. (C) Two-cell embryos were microinjected as indicated and allowed to develop to 22 hpf neurula. Whole-mount fluorescence immunocytochemistry was performed using an anti-tubulin-β 2B (TUBB2B) antibody. Representative neural plates are shown. TUBB2B staining intensity was measured on the two sides of the neural plate, and the intensity on the injected side was divided by the intensity on the uninjected side. Embryo numbers: n = 12 for GFP, n = 14 for NTF2, n = 10 for imp α + GFP-LB3. Error bars represent SD. NS not significant. (TIF) [file pone.0215740.s003.tif]

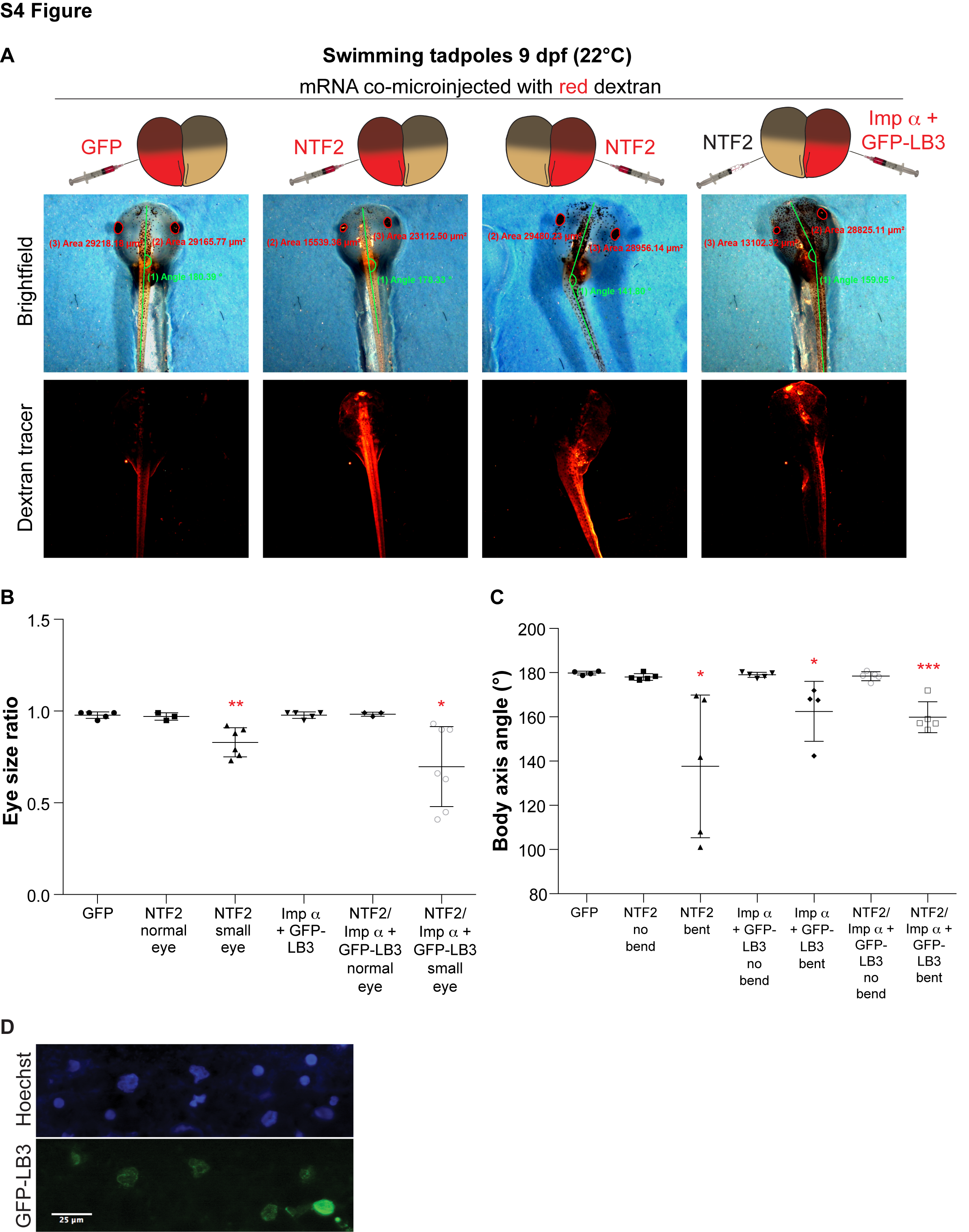

Supplement: S4 Fig — (A) Two-cell embryos were microinjected as indicated and allowed to develop into 9 dpf swimming tadpoles. Representative images are shown. Eye areas were measured from brightfield images, as shown in red text. Body axis bend angles were measured from brightfield images, as shown in green text. Note that the bent tadpole microinjected with NTF2 is the same one shown in Fig 3A. (B) For each tadpole, the area of the eye on the injected side was divided by the area of the eye on the uninjected side to obtain the eye size ratio. Average ratios are plotted for 3–7 tadpoles per condition. These ratios were compared to the GFP injected controls to identify tadpoles with small eyes on the injected side having eye size ratios less than 1. Error bars represent SD. ** p<0.01, * p<0.05. (C) Average body axis angles are plotted for 4–5 tadpoles per condition. Error bars represent SD. *** p<0.005, * p<0.05. (D) One blastomere of a two-cell embryo was microinjected with GFP-LB3 mRNA. The embryo was allowed to develop into a tadpole. After staining with Hoechst, nuclei in the tadpole tail were visualized as indicated. GFP-LB3 expression persists in the tadpole. Nuclei expressing GFP-LB3 are larger than nuclei in non-expressing neighboring cells, visible only by Hoechst-staining in the upper panel. These results are consistent with studies showing that protein expression from microinjected mRNA can persist up to several days [50, 51]. (TIF) [file pone.0215740.s004.tif]

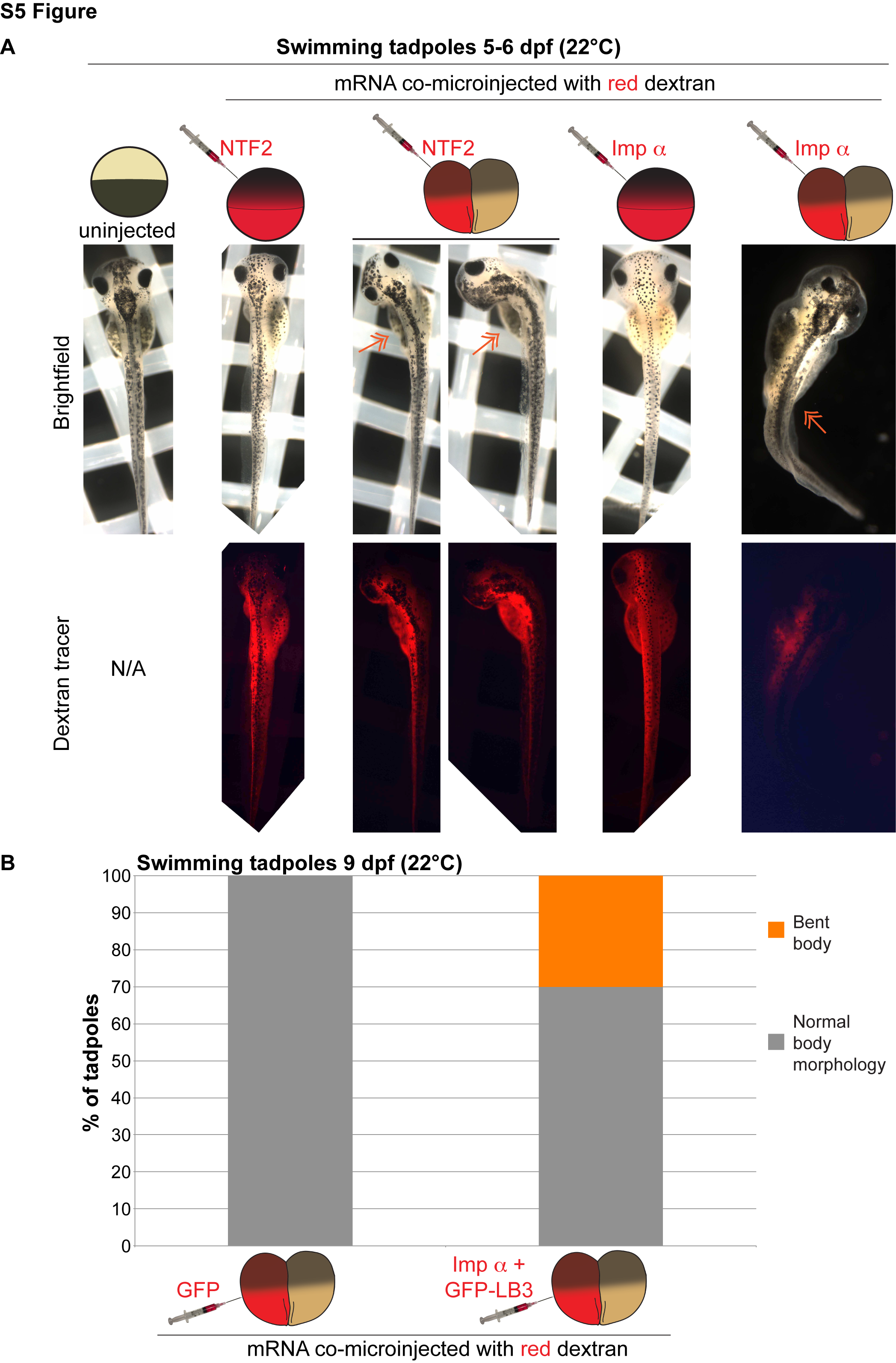

Supplement: S5 Fig — (A) Column 1: uninjected embryo. Column 2: One-cell embryo was microinjected with NTF2. Column 3: One blastomere of a two-cell embryo was microinjected with NTF2. Column 4: One-cell embryo was microinjected with importin α. Column 5: One blastomere of a two-cell embryo was microinjected with importin α. Embryos were allowed to develop into 5–6 dpf swimming tadpoles. Representative images are shown. Double-headed arrows indicate bent bodies. (B) Two-cell embryos were microinjected as indicated and allowed to develop into 9 dpf swimming tadpoles. Tadpoles were scored as indicated by measuring body axis angle. Embryo numbers: n = 10 for GFP, n = 10 for imp α + GFP-LB3. The GFP microinjection quantification is the same as shown in Fig 3B. Data presented in Fig 3 and S5 Fig were generated from two different frog colonies. (TIF) [file pone.0215740.s005.tif]

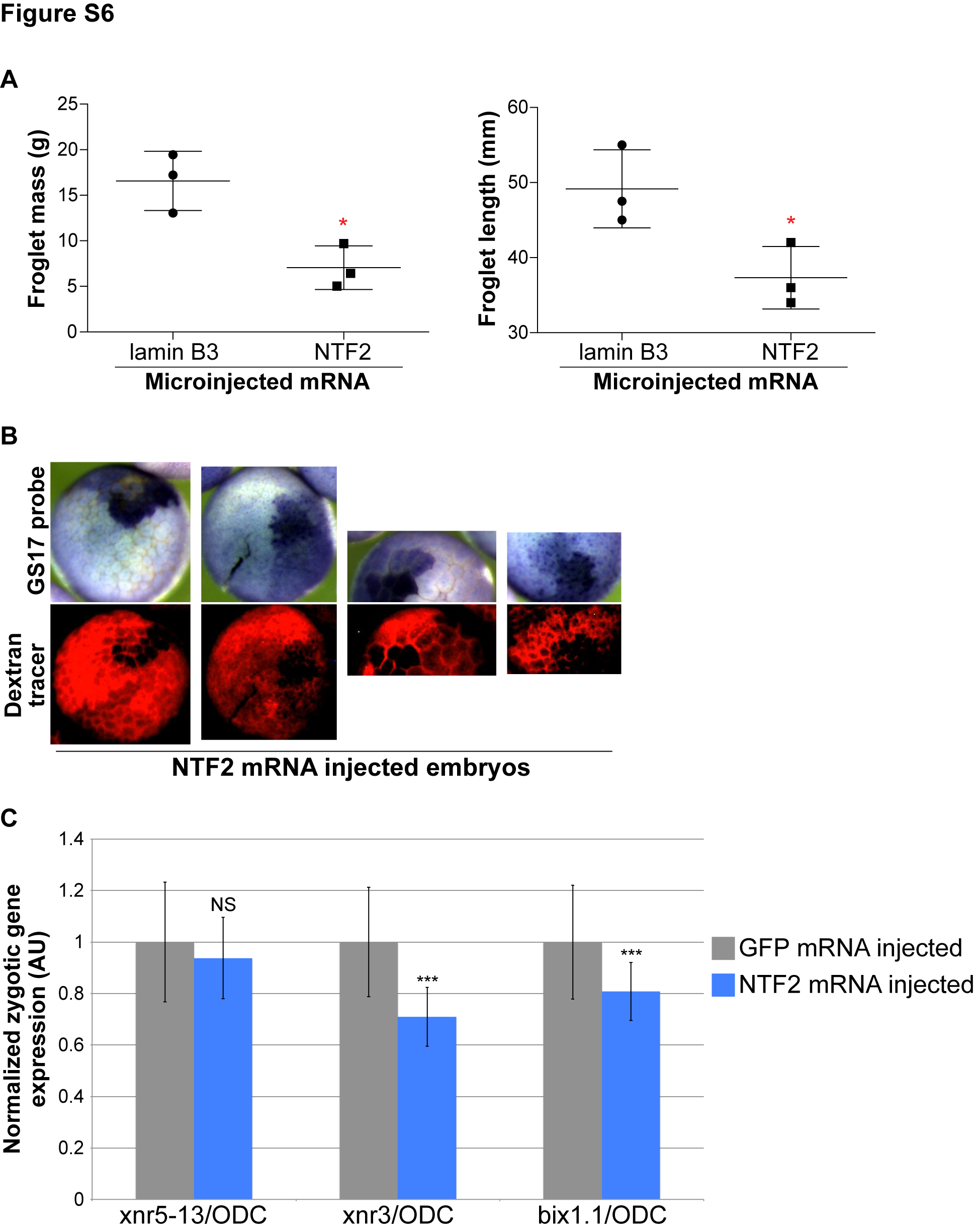

Supplement: S6 Fig — (A) At the two-cell stage one blastomere was microinjected with the indicated mRNA, and embryos were allowed to develop into froglets. Froglet body mass and length were quantified. Froglet numbers: n = 3 for lamin B3 and n = 3 for NTF2. (B) One blastomere of a two-cell embryo was co-microinjected with rhodamine-labeled dextran and NTF2 mRNA. Post-MBT embryos were subjected to in situ hybridization to detect the GS17 transcript. The top panels are bright-field images of embryos stained for GS17 (purple). The bottom panels are the corresponding rhodamine fluorescence images indicating cells in the embryo that received the NTF2 mRNA. Representative embryos are shown. (C) One-cell embryos were microinjected with GFP or NTF2 mRNA and allowed to develop to post-MBT (7.5 hpf). Total RNA was isolated from 12 embryos for each condition and converted to cDNA. Expression levels of three zygotic genes (xnr5-13, xnr3, and bix1.1) were determined by qPCR, normalized to ODC. Gene-expression levels are plotted in arbitrary units (AU) relative to GFP mRNA-injected control embryos. The means from 2 independent experiments are shown. Error bars represent SD. *** p<0.005, * p<0.05, NS not significant. (TIF) [file pone.0215740.s006.tif]
